# Plasmonic Ag@Cu_2_O core–shell nanostructures exhibiting near-infrared photothermal effect†

**DOI:** 10.1039/d3ra06712b

**Published:** 2023-10-27

**Authors:** Mariia Ivanchenko, Alison L. Carroll, Andrea B. Brothers, Hao Jing

**Affiliations:** a Department of Chemistry and Biochemistry, George Mason University Fairfax Virginia 22030 USA hjing2@gmu.edu; b Department of Chemistry, American University Washington DC 20016 USA

## Abstract

This work was devoted to the investigation of the optical properties, structural characterization, and photothermal conversion performance of Ag@Cu_2_O nanostructures. The selection of anisotropic silver core, specifically Ag nanocubes, was driven by the possibility to tune LSPR across a broader range of the electromagnetic spectrum. The thickness of the Cu_2_O shell was intentionally changed through the variation in the Cu salt to the metal core nanoparticles ratios. The LSPRs of Ag(nanocube)@Cu_2_O core–shell nanoparticles can be fine-tuned to the spectral region to become resonant with the excitation wavelengths of 808 nm NIR laser. Due to the high refractive index of the deposited Cu_2_O, the redshifts of the plasmon band wavelength in the extinction spectra were observed. Consequently, the photothermal activities of the Ag(nanocube)@Cu_2_O core–shell NPs have been controlled by the shell thickness at the nanoscale. Ag@Cu_2_O nanoparticles with thickest shell (∼70 nm) exhibit the most efficient NIR photothermal effect under the irradiation of 808 nm laser at ambient conditions. Results of this work demonstrate that Ag@Cu_2_O hetero-nanostructures may be optimized and used for the efficient transformation of light into other forms of energy, specifically heat.

## Abstract 2

Formation of Ag(nanocube)@Cu_2_O nanocomposite system offers several advantages, such as tunability of optical characteristics, that are pivotal to the enhancement of their photothermal properties.

## Graphical abstract

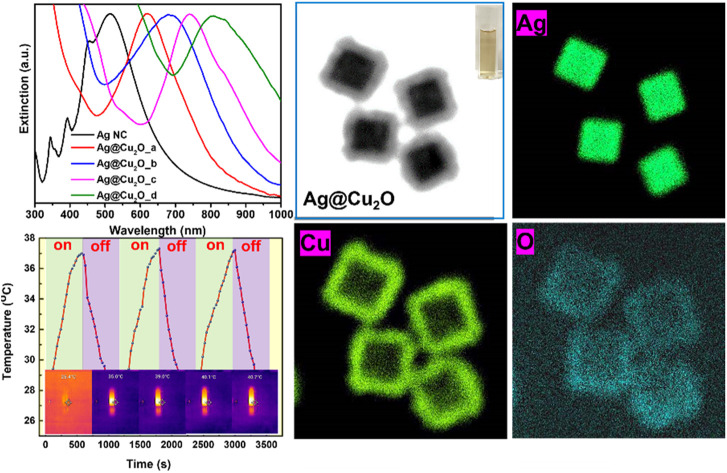

## Introduction

Attempts to obtain all-in-one nanomaterials with the ability to combine and tailor optical, electrical, magnetic, or catalytic functionalities, enhance charge and energy transfer, improve stability and durability led scientists to the discovery of numerous new nanostructures.^1,2^ An important type of such multicomponent nanosystems is formed by the integration of a noble metal with a semiconductor. This kind of hetero-nanostructures demonstrates enhanced and modifiable localized plasmon resonances (LSPRs) properties and facilitates efficient charge transfer through the interface, improving the overall charge carrier lifetime. Among others, Ag@Cu_2_O has been devoted a lot of attention due to various potential applications in plasmon-enhanced spectroscopy,^3,4^ photocatalysis,^5–8^ photovoltaics,^9,10^ biomedical applications,^11,12^ and bacterial contamination inhibition.^13,14^

Ag@Cu_2_O hetero-nanostructures offer a versatile platform for combining the unique properties of silver and Cu_2_O nanomaterials.^15^ Silver nanoparticles (NPs) possess stronger plasmon resonances compared to other noble metals^16^ and can act as more efficient light absorbents, capturing photons and transferring the energy to the semiconductor matrix, which is particularly useful for their applications in solar cells and solar fuel devices to enhance light absorption and improve overall device performance.^17^ Also, the photogenerated electrons from Cu_2_O can transfer to the Ag NPs due to their lower Fermi level, overcoming a Schottky barrier until equilibrium is reached and preventing the recombination of opposite charges.^18,19^

The precise control over the synthetic parameters, geometries, and interface engineering between Ag and Cu_2_O is vital for optimizing the properties of Ag@Cu_2_O nanostructures for specific applications.^20^ By precisely controlling the overall physical dimensions experimentally, the LSPRs of Ag nanocubes can be tailored across the visible and near-infrared (NIR) regions in the extinction spectra.^21^ Compared to their quasi-spherical counterparts, the plasmon resonances of the anisotropic Ag nanocubes are more sensitive to the refractive index of the surrounding medium.^22–24^ The plasmon tunability of the resulting heteronanostructures with Ag nanocubes and Cu_2_O as the core and shell, respectively can be maneuvered over a broader spectral range than the isotropic core–shell nanospheres of the similar core size and shell thickness.

Ag(nanocubes)@Cu_2_O nanostructures exhibit interesting NIR photothermal properties due to the combination of plasmonic properties of the cubic silver core and the light adsorption capability of the cuprous oxide shell. When LSPRs of Ag nanocubes are exited with the resonant wavelengths, the short-lived dephasing process results in a non-equilibrium distribution of the excited charge carriers within a few femtoseconds. Due to the scattering of these charge carriers, a Fermi distribution with energetic hot charge carriers is generated followed by a charge carrier–phonon interaction. The heat is then released to the surroundings through the final relaxation/dissipation *via* phonon–phonon scattering interaction.^25–28^ Also, the localized electromagnetic field enhancement near the surface of the nanocubes enhances light absorption and results in local temperature increase in the solution. On the other hand, the Cu_2_O component demonstrates strong light absorption in the visible range due to its direct bandgap, which can be converted into heat through various relaxations and electron–phonon interactions as well.^29^

The plasmon-mediated photothermal effect has several advantages due to the fast-heating rate and the possibility to adjust various properties of the plasmonic metal NPs or its composite to influence the heating efficiency. Deposition of Cu_2_O shell of various thicknesses on the Ag nanocubes progressively tunes LSPRs to NIR region or “biological window”. Utilization of NIR lasers to induce the photothermal effects with Ag@Cu_2_O heteronanostructures has significant implications in various biomedical applications owing to the superior tissue penetration of NIR light photons compared to visible light. For example, in photothermal therapy, these nanostructures can be selectively delivered to cancer cells and, upon NIR light irradiation, generate localized heat to induce hyperthermia, leading to the targeted destruction of cancer cells.^30,31^ Also, the photothermal effect provides a heating mechanism that may contribute to the enhanced photocatalytic performance of Ag@Cu_2_O core–shell nanostructures. Main advantage of using 808 nm NIR laser for the photothermal effects compared with other NIR excitations, such as most widely used 980 nm, is that 808 nm NIR light photons minimize the overheating effect due to their much lower absorption coefficients displayed by water molecules and biological tissues at this wavelength.^32–35^ Without being attenuated while passing through biological samples, the tissue penetrability is greatly improved using 808 nm NIR laser. Hence, the studies on the NIR photothermal effects induced by 808 nm laser using plasmonic Ag@Cu_2_O hybrid nanostructures with tunable LSPRs can positively expand the horizons of existing core–shell nanomaterials and have broad prospects for biological research relevant to human health.

Here, we report on the preparation of colloidal Ag(nanocube)@Cu_2_O nanostructures with optimized NIR photothermal properties. For the variety of the deposited shell sizes, nanostructures that exhibited plasmonic energy matching laser excitation wavelength demonstrated better heat generation performance. Notably, the LSPRs band of obtained Ag@Cu_2_O core–shell heteronanostructure with the densest shell thickness (∼70 nm) is fine-tuned to be resonant with the NIR laser (excitation wavelength of 808 nm) in the experimentally measured extinction spectra.

## Experimental

### Chemicals and materials

Silver trifluoroacetate (CF_3_COOAg, ≥99.99%), silver nitrate (AgNO_3_, ≥99.9999%), polyvinylpyrrolidone (PVP, average MW 55 000), hydrochloric acid (HCl, 37%), sodium hydrosulfide hydrate (NaHS·*x*H_2_O), acetone (99.6%), ethylene glycol (EG, 99.8%), copper(ii) nitrate hydrate (Cu(NO_3_)_2̇_·H_2_O, 99.999%), sodium hydroxide (NaOH, ≥98%), hydrazine (N_2_H_4_·3H_2_O, 35 wt% solution in water). All the chemicals used for the synthesis were of analytical grade and were used without further purification. The deionized water used in the experiments was ultra-pure (Milli-Q, 18 MΩ).

### Synthesis of ∼35 nm Ag seeds

In a typical synthesis, 20 mL of EG was added into a 50 mL flask and heated at 150 °C in an oil bath for 40 minutes under magnetic stirring (700 rpm). Next, 250 μL of freshly prepared 3 mM NaHS solution in EG, 2 mL of 3 mM HCl, 5 mL of 50 mg mL^−1^ PVP in EG, and lastly 1.5 mL of 282 mM CF_3_COOAg solution in EG were sequentially added into the flask. The flask was capped for the entirety of the process except during the additions of reagents and obtaining aliquots to control the reaction. The progress of the Ag seeds formation was monitored with UV-vis spectroscopy and the reaction was stopped using an ice-water bath when the LSPR peak was centered at ∼425 nm. After being washed at 8000 rpm for 8 minutes with acetone and DI water, the seeds were redispersed in 5 mL of EG for storage and used for further growth.

### Growth of ∼80 nm Ag nanocubes

To synthesize Ag nanocubes, 10 mL of EG was added into a 50 mL flask and heated at 150 °C in an oil bath for 10 minutes under magnetic stirring (1000 rpm). Then, 3 mL of 250 mg mL^−1^ PVP solution in EG was added and stirred for another 10 minutes. Then, 200 μL of the pre-formed Ag seeds were introduced, followed by the dropwise addition of 2 mL of 282 mM AgNO_3_ EG solution. Ag nanocubes with a side length of ∼80 nm were obtained by quenching the reaction in an ice-water bath when the LSPR peak of the product red-shifted to ∼500 nm. The product was washed with acetone and water twice and then redispersed in 2 mL of EG for further use.

### Formation of Ag@Cu_2_O core–shell heteronanostructures

To epitaxial deposit Cu_2_O shells onto Ag nanocubes, 100 μL of colloidal Ag nanocubes was first introduced into 5 mL of 2 wt% PVP aqueous solution. 10, 20, 30, or 40 μL of 0.1 M Cu(NO_3_)_2_ aqueous solution were subsequently added. Then, 11.2 μL of 5 M NaOH and 5 μL of N_2_H_4_ solution were added under magnetic stirring (1200 rpm). The reaction was allowed to proceed for 10 min at room temperature and the resulting core–shell nanoparticles were washed with water.

### Methods and instrumentation

Optical extinction spectra were recorded using a Shimadzu UV-2600 Plus spectrophotometer. Transmission electron micrographs (TEM) were recorded using a JEOL JEM-1400Flash microscope operating at 100 kV. High-resolution transmission electron microscopy (HRTEM) images were collected utilizing a JEM 2100Plus TEM operating at 200 kV. Samples were coated onto a Ni grid to avoid the interference of peaks from the Cu grid and fixed on a JEOL single tilt beryllium retainer for energy-dispersive X-ray spectroscopy (EDS) analysis. Elemental mapping was performed on a JEM 2100Plus TEM with a JED-2300T EDS detector. The surface morphology of the NPs was analyzed using a JEOL JSM-IT500HR scanning electron microscope (SEM) operating at 15 kV.

### Photothermal measurements

The photothermal effects of Ag nanocubes and Ag@Cu_2_O with various semiconductor shell thicknesses were evaluated using 808 nm laser. Briefly, nanoparticles were suspended in 1.5 mL of water (∼11.6 mM of Ag) in 1 cm long with 4 transparent side quartz cuvette and were irradiated with 2.0 W cm^−2^ continuous NIR laser (MDL-H-808-5W, Changchun New Industries Optoelectronics Tech Co, Ltd, China) for 20 minutes. The temperature in each sample was recorded every 5 minutes by an infrared (IR) thermal imaging camera (TG165-X, FLIR, Taiwan). Also, the Ag@Cu_2_O_d suspension stability was tested irradiating the sample with an 808 nm for 10 min and allowing it to cool down when laser was turned off for another 10 minutes for 3 cycles.

### Calculation the photothermal conversion efficiency

The photothermal conversion efficiency was calculated using a macroscopic model of collective particle heating describing heat generation and dissipation of the NPs suspension.^36^ The temperature *T* change with time *t* is described by the energy balance equation1
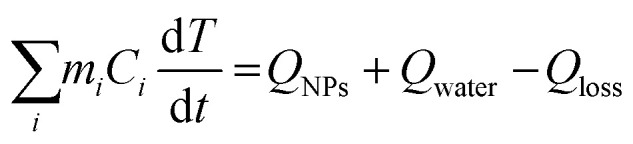
where *Q*_NPs_ is the heat induced by the absorption of the nanoparticles, *Q*_water_ is heating of the solvent, *Q*_loss_ is the heat dissipated by the solvent, *m*_*i*_ and *C*_*i*_ are the mass and specific heat capacities of each element in the system. The contributions of the NPs can be considered negligible because the mass of hetero-nanostructures was small compared to the mass of the solvent (1.5 g), and the heat capacity of silver (0.233 J g^−1^ K^−1^) and Cu_2_O (0.297 J g^−1^ K^−1^) are much smaller than that of water (4.184 J g^−1^ K^−1^). Then, the absorption and dissipation energy can be presented as

2 *Q*_NPs_ = *P*(1 − 10^−*A*_λ_^)*η*

3 *Q*_loss_ = *hS*(*T* − *T*_room_)

where *A*_*λ*_ is the optical extinction at the incident wavelength, *P* is the power of the excitation laser, *η* is the conversion efficiency, *h* is the heat transfer coefficient, *S* is the cross-section surface area of thermal convection of the solution to the air.

A dimensionless driving force temperature, *θ*, is introduced as4
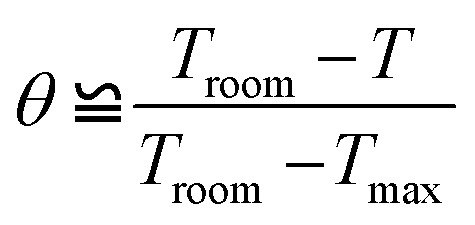
where *T*_room_ is the room temperature and *T*_max_ is the maximum temperature reached under illumination. The thermal equilibrium time constant (*τ*) could be obtained by fitting the experimentally measured temperature change of the solution during cooling periods using the linear equation5
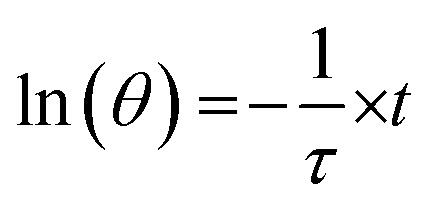


*τ* provides information on how fast the system loses energy to the environment (Fig. S5, ESI†). The heat-transfer coefficient can be estimated as6
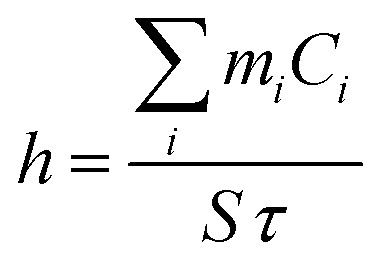


When system is at equilibrium

7 *Q*_loss_ = *Q*_NPs_ + *Q*_water_

The *Q*_water_ was obtained from the temperature changes of the pure solvent8
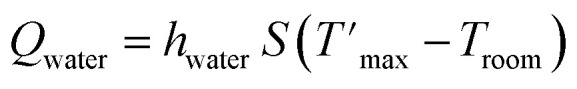


Expressing *Q*_NPs_ = *Q*_loss_ − *Q*_water_ and substituting it in eqn (2), the final expression for the photothermal conversion efficiency appears in the following form9
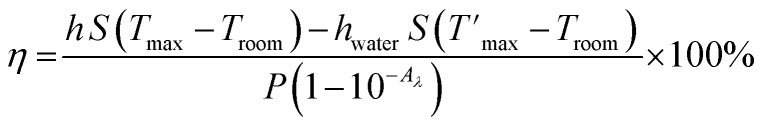


## Results and discussion

**Fig. 1:**
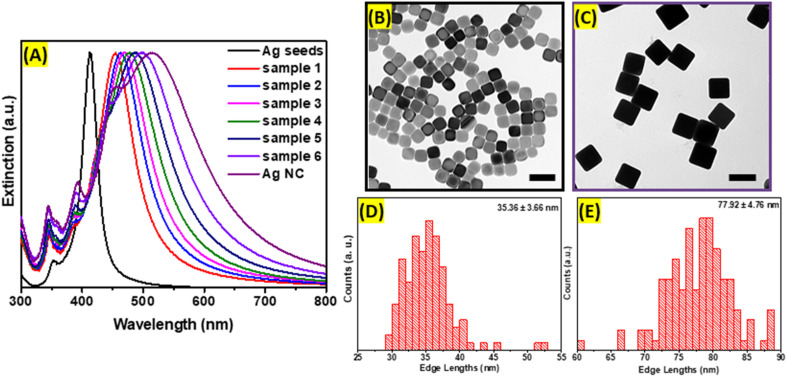
(A) Extinction spectra measured during the seed-mediated overgrowth of Ag nanocubes. TEM images of (B) cubic Ag seeds and (C) large Ag cubes. Scale bars: 100 nm. Size distributions of the edge lengths of (D) cubic Ag seeds and (E) large Ag cubes.

Ag nanocubes were obtained using the seed-mediated method, which allows precise control of the size distribution and shape purity. First, seeds were formed using polyol as a solvent and reducing agent at the elevated temperature required to initiate the reduction process of silver precursor. The polyol, specifically ethylene glycol, prevents rapid growth of the nuclei into large nanoparticles, while PVP serves as a capping agent preserving (100) planes and forming cubic shape. The extinction spectrum of Ag nanoseeds exhibits a distinct dipolar plasmonic resonance peak centered at 420 nm. In addition to the main LSPRs peak, seeds possess a higher energy plasmonic mode evidenced by the presence of the small peak positioned at around 360 nm corresponding to an octupole plasmon mode.^20^ As shown in the TEM image (Fig. 1B), the obtained Ag seeds are uniform and have well-defined cubic geometry with slightly rounded corners. The mean edge length of synthesized seeds is 35.36 nm with a standard deviation of 3.66 nm, as demonstrated in the size distribution histogram (Fig. 1D). Second, larger Ag nanocubes were grown after injection of pre-farmed seeds into the growth solution containing AgNO_3_ in ethylene glycol at 150 °C. Ag^+^ ions were reduced and preferably deposited onto the seeds' surfaces. The process was monitored in real time by periodically sampling the reaction mixture and analyzing it with extinction spectroscopy as demonstrated in Fig. 1A. The growth was terminated when the dipolar LSPRs peak redshifted 500 nm. During the process, several new extinction peaks emerged corresponding to higher-order modes and were assigned to the dipole, quadrupole, octupole, and higher-order multipole plasmon resonances, respectively, in the order from longer to shorter wavelengths in the spectrum (Fig. 1A).^20^ Highly monodisperse Ag nanocubes with average edge lengths of 77.92 nm (±4.76 nm) were obtained and cubic morphology with slight truncations at the corners were observed in TEM images (Fig. 1E).

**Fig. 2:**
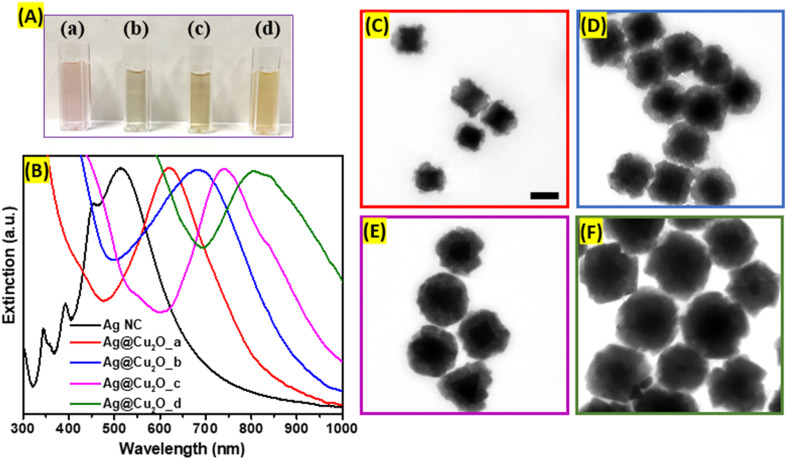
(A) Digital photographs of Ag@Cu_2_O hybrid nanoparticles colloidal solutions synthesized adding (a) 10 μL, (b) 20 μL, (c) 30 μL, (d) 40 μL of 0.1 M Cu(NO_3_)_2_. (B) Extinction spectra of colloidal solutions of Ag@Cu_2_O hybrid nanoparticles with varying shell thicknesses. TEM images of Ag@Cu_2_O hybrids synthesized adding (C) 10 μL, (D) 20 μL, (E) 30 μL, (F) 40 μL of 0.1 M Cu(NO_3_)_2_. Scale bar: 100 nm.

Ag@Cu_2_O hybrid nanostructures were formed after the epitaxial deposition of Cu_2_O layers on the surfaces of large Ag nanocubes. The deposition of Cu_2_O shell redshifts the Ag nanocubes plasmon resonance peak centered at 510 nm to 620 nm, 685 nm, 745 nm, 805 nm with increase of the thickness, which is attributed to the changes in the charge density oscillations induced by the presence of copper(i) oxide and the increase in refractive index of the shell (Fig. 2B). This type of nanostructure combines the plasmonic properties of Ag core with the unique optical and electronic characteristics of Cu_2_O shell. The optical features of such nanocomposites could be tailored by controlling the size of the cubic core and/or shell to suit prospective application requirements. Therefore, Ag@Cu_2_O hetero-nanostructures with different shell thicknesses were synthesized by adjusting the concentrations of Cu-precursor to precisely tune the extinction peak across visible and NIR regions in the extinction spectrum. The spectral features at the higher energy for all Ag@Cu_2_O colloidal solutions are attributed to the inter-band transitions in the Cu_2_O shell. The corresponding color change of Ag@Cu_2_O colloids from purple to dirty yellow was observed. Micrographs showed individual, well-separated nanostructures with distinct core–shell arrangements. The silver core appeared as a well-defined, electron-dense region at the center, while the Cu_2_O shell surrounded the core, forming a continuous and dense layer (Fig. 2C–F). The average Cu_2_O shell thicknesses were measured to be 13.7, 29.6, 40.1, and 71.0 nm (Fig. S1, ESI†). For the Ag@Cu_2_O sample with the thinnest shell, Cu_2_O size varied greatly, and different crystalline domains were coating the Ag core. As the shell thickness of Ag@Cu_2_O increased, the contrast between the core and shell in TEM images disappeared. SEM images revealed rough outer surfaces and irregular faceting of the hybrid nanoparticles (Fig. S2, ESI†).

**Fig. 3:**
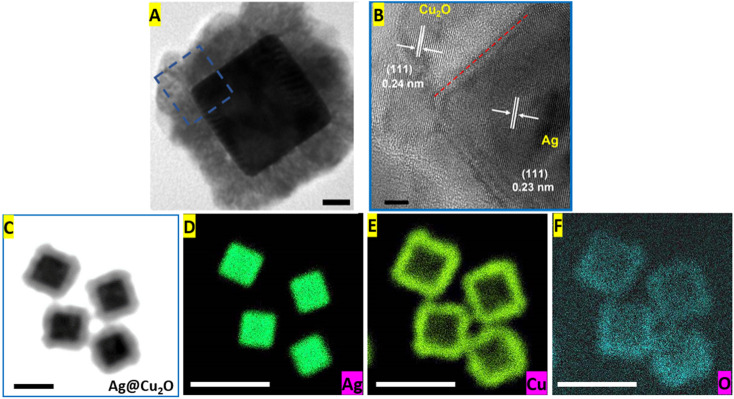
(A) HRTEM images of an Ag@Cu_2_O nanoparticle synthesized using 20 μL of 0.1 M Cu(NO_3_)_2_ with denoted interface area that was examined. Scale bar: 20 nm. (B) HRTEM image of an Ag@Cu_2_O nanoparticle with indicated interplanar spacings corresponding to the lattice fringes of the (111) planes of the Ag core and Cu_2_O shell. Scale bar: 5 nm. (C) TEM image of Ag@Cu_2_O hybrids synthesized adding 20 μL of 0.1 M Cu(NO_3_)_2_ and used for EDS mapping. Scale bar: 100 nm. Elemental distributions of (D) Ag, (E) Cu, and (F) O in Ag@Cu_2_O nanoparticles. Scale bars: 200 nm.

More advanced techniques, such as HR-TEM and EDS elemental mapping, were used to obtain additional details about the structural and compositional characteristics of the Ag@Cu_2_O core–shell nanostructures. The lattice fringes of both components were visible in the HR-TEM image of the Ag@Cu_2_O nanoparticle (Fig. 3A and B). The spacing of these lattice fringes of the Ag nanocube core was 0.23 nm, close to the *d* value of the Ag (111) plane, and the lattice parameter measured from the fringes of Cu_2_O was 0.24 nm corresponding to the fcc Cu_2_O (111) plane. Also, the alignment of the fringes in the same direction confirmed the epitaxial growth of the shell materials on the Ag core. Elemental mapping images provided visualization of the spatial distribution of silver, copper, and oxygen elements within the Ag@Cu_2_O hybrid nanostructure (Fig. 3C–F). The silver core showed a high concentration of Ag element represented by a dense accumulation of high-intensity signals in the corresponding map (Fig. 3D). Signals of the Cu and O elements appeared in the hybrids' outer layers, leaving very few low-intensity signals inside. It is noteworthy to mention that the copper map had more pronounced signals than the elemental distribution image of oxygen because the concentration of Cu present in the shell was twice of O-element concentration (Fig. 3E and F).

**Fig. 4:**
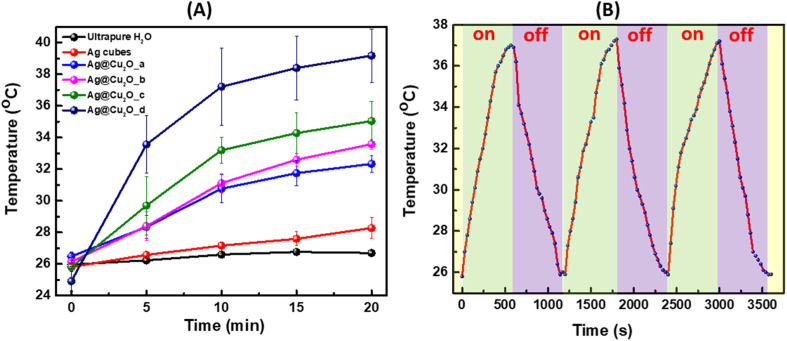
(A) Temperature changes of Ag nanocubes, Ag@Cu_2_O hybrid nanoparticles colloidal solutions synthesized adding (a)10 μL, (b)20 μL, (c) 30 μL, (d) 40 μL of 0.1 M Cu(NO_3_)_2_ and water over time under irradiation with the NIR laser at 808 nm. (B) Photothermal stability test of Ag@Cu_2_O_d exposed to 808 nm laser irradiation for three cycles.

During laser illumination of Ag@Cu_2_O nanostructures suspended in water, the light absorbed by the nanoparticles is converted into heat due to a nonradiative relaxation process, which leads to a rise in local temperature and transfer of generated heat to the surrounding medium. We would like to emphasize that the shell will not hinder the heat dissipation because nanostructures are well dispersed in water, and the Cu_2_O shell is less dense structure than traditional solid Cu_2_O allowing heat to be transmitted through the shell. The experimental setup for the photothermal effect measurements is provided in Fig. S3, ESI.† The results were presented as a plot of temperature rise *versus* time of irradiation. Fig. 4A showed an initial rapid temperature increase that then slowed down for all Ag@Cu_2_O samples. Expectedly, colloids with extinction peaks positioned closer to or resonant with the excitation wavelength of the laser possessed larger photothermal conversion efficiency, which resulted in more distinct temperature rises. The temperature elevated for an average of 5.8, 7.4, 9.3, and 14.3° for the Ag@Cu_2_O colloids as their shell thickness increased under the excitation of 808 nm laser as demonstrated in Fig. 4A. The 808 nm laser has gained significant importance and is actively utilized as an excitation source in biomedical applications due to its safety for biological tissues, minimal phototoxicity, low-energy non-ionizing radiation range, and deep tissue penetration due to the position within “biological window” of tissue transparency minimizing absorption and scattering. Corresponding IR camera pictures of the four samples with indicated measured temperatures for selected trials are presented in Fig. S4, ESI.† To validate the occurrence of the photothermal effect and evaluate the specific contribution of Ag@Cu_2_O, control measurements were performed for the suspension of bare Ag nanocubes (77.92 nm ± 4.76 nm) with identical concentration and pure water under the same experimental conditions. There was no change in temperature for pure water with 808 nm laser excitation for 20 min at ambient condition when reaching the thermal equilibria. Negligible temperature elevation (less than 3°) produced by colloidal Ag nanocubes was observed, demonstrating that the Ag@Cu_2_O core–shell nanostructures with LSPRs in NIR spectral region exhibit the most intense and efficient NIR photothermal effect. The photothermal conversion efficiency was calculated to be ∼25.4% for Ag@Cu_2_O with shell thickness of ∼70 nm and LSPRs at 805 nm. We used the extinction values to calculate photothermal efficiency as described in several previously published works.^37,38^ Although only absorption will contribute to the photothermal transduction, it is impossible experimentally distinguish between absorption and scattering relative contributions to the extinction. Also, it is worth noting that although scattering increases with the size of the nanostructure, absorption increases as well because of LSPR shifts closer to excitation wavelength of the laser, which contributes to higher temperature rises. Additionally, we would like to mention that there is room for the further improvement of photothermal efficiencies through structural engineering of hybrid hetero nanostructures. However, it is meaningless to directly compare the photothermal efficiency values of our samples to those of other nanoparticles or nanomaterials reported previously in the literature because the photothermal efficiency values are sensitively dependent upon the sizes, shapes, and compositions of the nanoparticles, the light absorption and scattering properties, the excitation wavelengths, and the local environments for heat dissipation, all of which varied drastically from case to case. Considering that LSPR response of the nanostructures was tuned to the excitation wavelength of the laser, further optimization of photothermal conversion efficiency under given conditions is possible by increasing concentration of the Ag@Cu_2_O nanostructures is suspension to a certain “critical” concentration where the aggregation occurs. For example, the photothermal transduction efficiency of Au nanorods has been previously reported in the 20–30% range,^39,40^ which is about the same as of discussed Ag@Cu_2_O nanostructures. However, the composition of those NPs is different and gold has a different work function compared to silver and we do not think it would be appropriate to compare different materials from this viewpoint. Moreover, the transduction efficiency depends on size, structure, illumination density, concentration, which need to be accounted for during the comparison.

Then, the temperature change of the Ag@Cu_2_O suspension with time was monitored upon irradiation with 808 nm laser for 10 minutes with subsequent natural cooling for the same time when laser was turned off. The photothermal stability was tested for three cycles as presented in Fig. 4B, showing no obvious difference after on/off cycles were complete.

## Conclusions

In summary, Ag@Cu_2_O hybrid NPs were successfully fabricated in a three-step approach. First, the cubic Ag core nanocrystals with sizes of ∼80 nm were prepared by growing silver over Ag seeds. Second, the growth of Cu_2_O shells of various thicknesses was performed at room temperature in water. Obtained heteronanostructures exhibit optical properties from both the Cu_2_O shells and the Ag cores. The Ag LSPR extinction band position changes with the shell thickness. By combining noble metal and semiconductor shell, hybrid nanostructure with optical response in NIR region can be designed *via* wet chemistry approach. Compared to other traditionally used semiconductor materials, such as SiO_2_ and TiO_2_ shells, only Cu_2_O shell permits to modulate LSPR to NIR region.^41^ When Ag(nanocube)@Cu_2_O extinction band deviated from the laser wavelength at 808 nm, the photothermal effect diminished resulting in small or negligible temperature changes. As the LSPR band approaches that of the excitation wavelength, colloidal Ag@Cu_2_O samples demonstrate excellent photothermal performance with a temperature rise of ∼15 degrees Celsius in 20 minutes of continuous laser irradiation. Also, Ag@Cu_2_O hetero-nanostructures with ∼70 nm semiconductor shells more efficiently convert the absorbed light energy into heat through a nonradiative decay process than their counterparts with thinner shells or bare Ag nanocubes.

## Author contributions

Hao Jing conceived and designed the research. M. I. and A. L. C. synthesized the Ag(nanocube)@Cu_2_O nanoparticles, collected the extinction spectra and photothermal data. M. I. used scanning (JEOL JSM-IT500HR) and transmission electron microscopy (JEOL JEM-1400Flash) to characterize all nanostructures. A. B. B. obtained high-resolution and STEM elemental mapping images on all nanoparticles using JEM 2100Plus TEM equipped with JED-2300T EDS detector. Hao Jing supervised the research. Hao Jing and M. I. co-wrote the paper. All authors discussed the results and commented on the manuscript.

## Conflicts of interest

The authors declare no competing financial interest.

## Supplementary Material

RA-013-D3RA06712B-s001
