# Supplementary material for: Plasmonic Ag@Cu2O core–shell nanostructures exhibiting near-infrared photothermal effect
Source: RSC Adv. 2023 Oct 27;13(45):31569–77. doi: 10.1039/d3ra06712b (PMC10606979; doi:10.1039/d3ra06712b)
Supplement: RA-013-D3RA06712B-s001 [file RA-013-D3RA06712B-s001.pdf]

Electronic Supplementary Material (ESI) for RSC Advances.
This journal is © The Royal Society of Chemistry 2023

Plasmonic Ag@Cu_2O Core-Shell Nanostructures Exhibiting Near-Infrared Photothermal Effect

Mariia Ivanchenko,[†] *Alison L. Carroll,*[†] *Andrea B. Brothers,*[§] *and Hao Jing*[†, *]

[†] Department of Chemistry and Biochemistry, George Mason University, Fairfax, Virginia 22030, USA

[§] Department of Chemistry, American University, Washington, DC 20016, USA

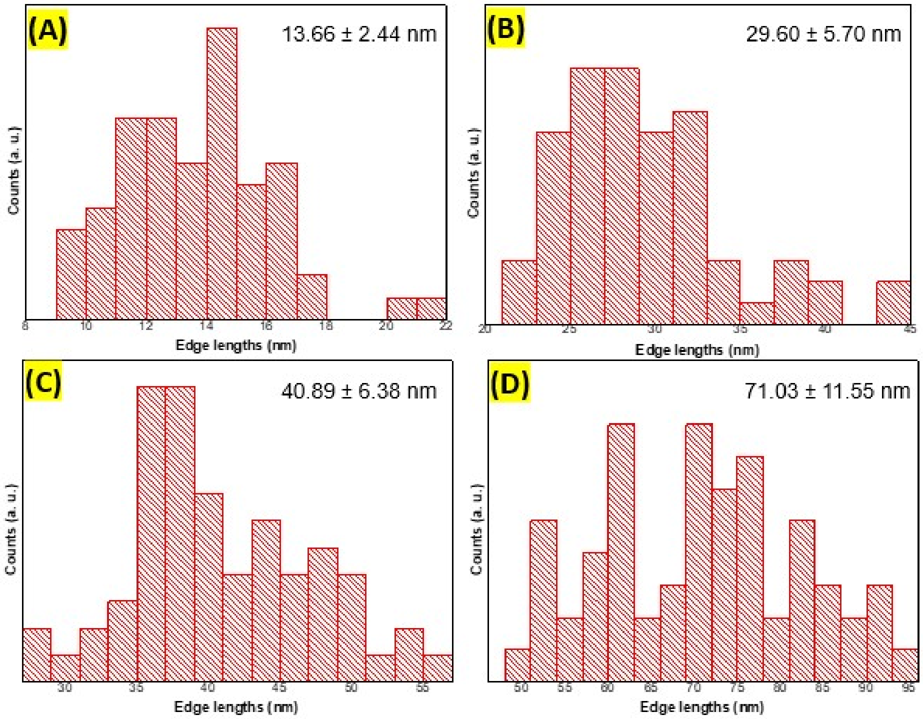

Fig. S1. Distribution of Cu_2O shell thickness measured from TEM images of Au@Cu_2O NPs synthesized using (A) 10 µL, (B) 20 µL, (C) 30 µL, (D) 40 µL of 0.1 M $Cu(NO_3)_2$.

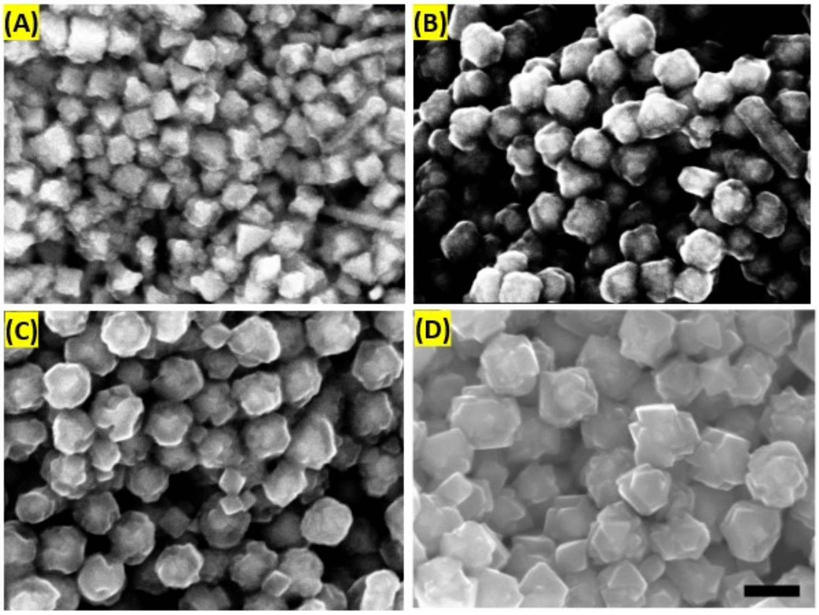

Fig. S2. SEM images of $Ag@Cu_2O$ nanoparticles obtained using (A) 10 μL, (B) 20 μL, (C) 30 μL, (D) 40 μL of 0.1 M $Cu(NO_3)_2$.

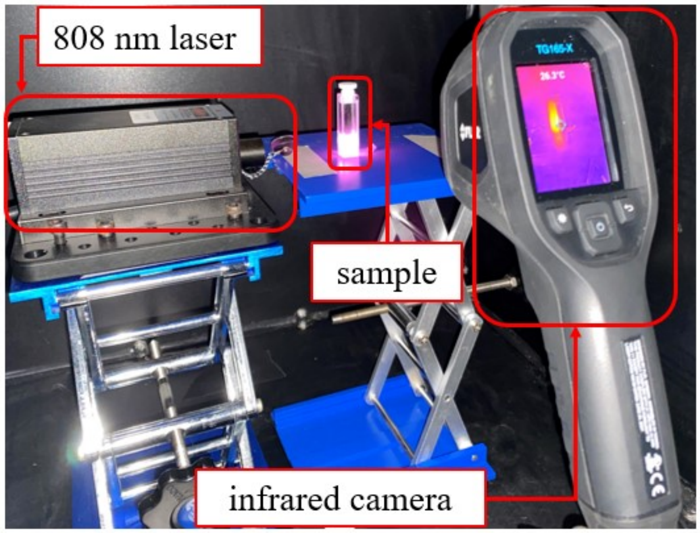

Fig. S3. Photothermal Heating Set Up.

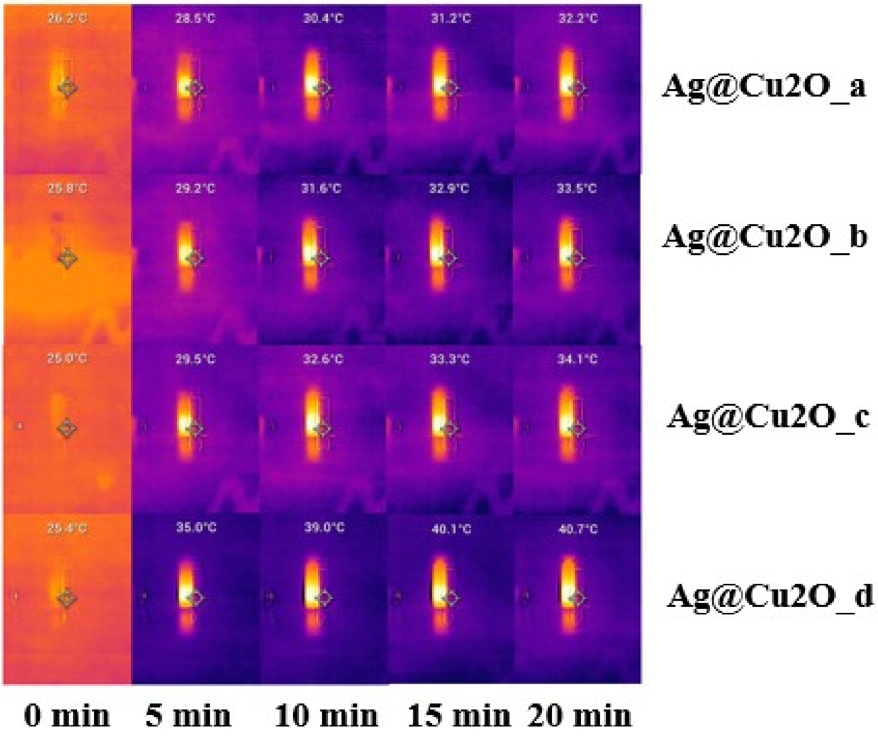

Fig. S4. Infrared thermal images showing temperature measurements of Ag@Cu_2O colloidal solutions synthesized adding (a)10 μL, (b)20 μL, (c) 30 μL, (d) 40 μL of 0.1 M $Cu(NO_3)_2$ under continuous 808 nm laser illumination.

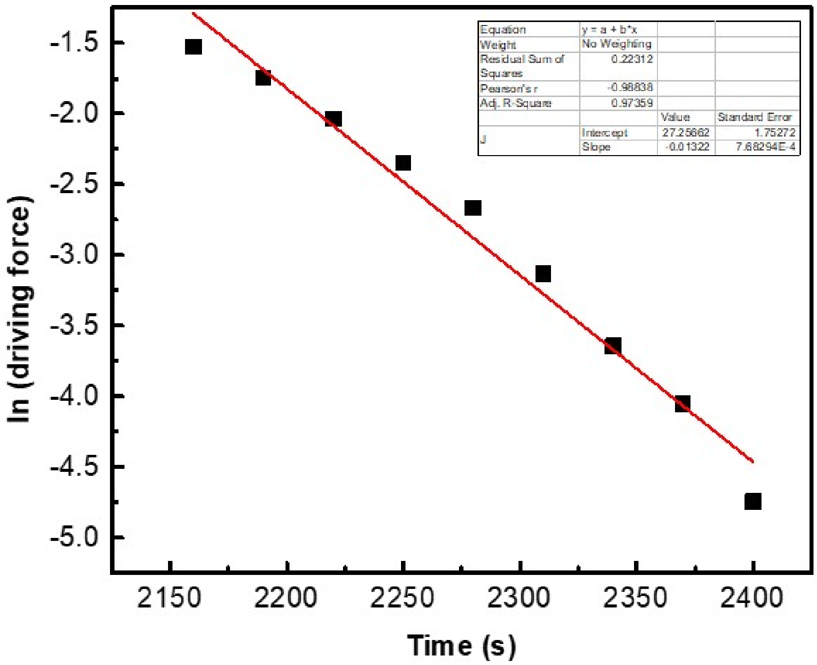

Fig. S5. The linear regression plot of the natural logarithm of driving force temperature against time used for calculation of the photothermal efficiency.
